# Defining the Functional Domain of Programmed Cell Death 10 through Its Interactions with Phosphatidylinositol-3,4,5-Trisphosphate

**DOI:** 10.1371/journal.pone.0011740

**Published:** 2010-07-23

**Authors:** Christopher F. Dibble, Jeremy A. Horst, Michael H. Malone, Kun Park, Brenda Temple, Holly Cheeseman, Justin R. Barbaro, Gary L. Johnson, Sompop Bencharit

**Affiliations:** 1 Department of Pharmacology, School of Medicine, and the Lineberger Comprehensive Cancer Center, University of North Carolina, Chapel Hill, North Carolina, United States of America; 2 Department of Prosthodontics and the Dental Research Center, School of Dentistry, University of North Carolina, Chapel Hill, North Carolina, United States of America; 3 Department of Microbiology, School of Medicine, and Department of Oral Biology, School of Dentistry, University of Washington, Seattle, Washington, United States of America; Griffith University, Australia

## Abstract

Cerebral cavernous malformations (CCM) are vascular abnormalities of the central nervous system predisposing blood vessels to leakage, leading to hemorrhagic stroke. Three genes, Krit1 (CCM1), OSM (CCM2), and PDCD10 (CCM3) are involved in CCM development. PDCD10 binds specifically to PtdIns(3,4,5)P3 and OSM. Using threading analysis and multi-template modeling, we constructed a three-dimensional model of PDCD10. PDCD10 appears to be a six-helical-bundle protein formed by two heptad-repeat-hairpin structures (α1–3 and α4–6) sharing the closest 3D homology with the bacterial phosphate transporter, PhoU. We identified a stretch of five lysines forming an amphipathic helix, a potential PtdIns(3,4,5)P3 binding site, in the α5 helix. We generated a recombinant wild-type (WT) and three PDCD10 mutants that have two (Δ2KA), three (Δ3KA), and five (Δ5KA) K to A mutations. Δ2KA and Δ3KA mutants hypothetically lack binding residues to PtdIns(3,4,5)P3 at the beginning and the end of predicted helix, while Δ5KA completely lacks all predicted binding residues. The WT, Δ2KA, and Δ3KA mutants maintain their binding to PtdIns(3,4,5)P3. Only the Δ5KA abolishes binding to PtdIns(3,4,5)P3. Both Δ5KA and WT show similar secondary and tertiary structures; however, Δ5KA does not bind to OSM. When WT and Δ5KA are co-expressed with membrane-bound constitutively-active PI3 kinase (p110-CAAX), the majority of the WT is co-localized with p110-CAAX at the plasma membrane where PtdIns(3,4,5)P3 is presumably abundant. In contrast, the Δ5KA remains in the cytoplasm and is not present in the plasma membrane. Combining computational modeling and biological data, we propose that the CCM protein complex functions in the PI3K signaling pathway through the interaction between PDCD10 and PtdIns(3,4,5)P3.

## Introduction

Cerebral cavernous malformations (CCM) are congenital or sporadic vascular disorders of the central nervous system (CNS) [1]–[17]. Prevalence ranges from 0.5 percent in the general population to 1.5 percent in Hispanics [2]–[10], [14]–[17]. Histopathologically, CCM are abnormally large harmatomous vascular lesions formed by a single layer of capillary endothelial cells without the support of brain parenchyma [1]–[2], [18]–[22]. Ruptured CCM lesions can cause hemorrhagic stroke and are often associated with seizures, recurrent headaches, and focal neurological defects (2–4). Three CCM loci have been mapped in humans: 7q21–22 (Krit-1 or CCM1), 7p13–15 (OSM or CCM2), and 3q25.2–27 (PDCD10 or CCM3). Mutations in these CCM loci cause loss of function of these proteins and result in CCM [7]–[16], [17], [23].

CCM3, the smallest of the CCM proteins, is a 25 KDa protein composed of 212 amino acids. It was originally identified as TF-1 cell apoptosis related gene-15 (TFAR15), since it is up-regulated with the induction of apoptosis by serum withdrawal in TF-1 human premyeloid cells [17], [23]. It was subsequently renamed PDCD10 (programmed cell death 10) as it was thought to be involved in apoptotic responses [17], [23]. PDCD10 is the third and latest CCM gene identified [17], [23]–[24]. The N-terminal region of PDCD10, which in some CCM patients is the site of an in-frame deletion of an entire exon encoding from L33 to K50, was found to be the binding site for the oxidant stress response serine/threonine kinase 25 (STK25) and the mammalian Ste20-like kinase 4 (MST4) [25]. Similar to earlier observations, PDCD10 was found to function in apoptotic pathways since overexpression of PDCD10 induces apoptosis through the caspase 3 pathway [26]. Furthermore, PDCD10 may be regulated through phosphorylation and dephosphorylation, since it can be phosphorylated by STK25 and dephosphorylated by binding to the phosphatase domain of Fas-associated phosphatase-1 [27]. Recently, we showed that all three CCM proteins (Krit1, OSM, and PDCD10) form a complex in the cell and that PDCD10 binds directly to OSM independently of the OSM-Krit1 interaction [28]. We also showed that PDCD10 binds to both phosphatidylinositol bis- or tris-phosphates, but seems to have the highest affinity to phosphatidylinositol-3,4,5-trisphosphate (PtdIns(3,4,5)P_3_) [28]. However, it is not known which part of PDCD10 binds to PtdIns(3,4,5)P_3_ or OSM because there is currently no structural data available for PDCD10. Creating a structural model of PDCD10 is, therefore, a critical first step to provide insight into the PDCD10 structure-function relationship. The interaction of PDCD10 and PtdIns(3,4,5)P_3_ suggests that PDCD10 may function in concert with phosphatidylinositol-3-kinase (PI3K), the enzyme that catalyzes the formation of PtdIns(3,4,5)P_3_ at the plasma membrane [29]. PI3K activation by growth factors including vascular endothelial growth factor (VEGF) is known to be crucial in angiogenesis. Thus, a relationship between PDCD10 and PI3K would be evidence that CCM development may result from dysregulation in the PI3K pathway through PDCD10-PtdIns(3,4,5)P_3_ interaction. In this study we attempted to define the functional domain of PDCD10 that is important in PtdIns(3,4,5)P_3_ binding by using molecular modeling combined with site-directed mutagenesis.

Homology modeling allows for identification of critical amino acid residues for protein-protein interaction and protein-ligand interaction [30]. The usefulness of homology is inversely dependent on the evolutionary distance between the target and templates [31]. The structural conservation between the target and template, as well as the correctness of the template alignment, are among the most important factors in generating homology models. Generating a homology model for PDCD10 is therefore challenging because of the low structural conservation with available templates and low sequence identity between PDCD10 and the templates [31]–[33]. Our homology models and biophysical data predict that PDCD10 is likely a dimeric six-helical protein composed of two trihelical heptad-repeat structures. We identified the amphipathic helix and Lys residues essential for PDCD10-PtdIns(3,4,5)P_3_ and PDCD10-OSM interaction. Finally, we demonstrated that using membrane-bound constitutively active PI3K (p110-CAAX), the wild-type (WT) PDCD10 co-localizes with the p110-CAAX mostly at the plasma membrane, while the mutants lacking Lys residues remain in the cytoplasm.

## Results

### Threading analysis and homology modeling of PDCD10

The structure of PDCD10 was first examined using 3D-Jury to generate meta-predictions for PDCD10 [34]. Unlike Krit1 or OSM, which are large scaffolding proteins composed of multiple functional domains, PDCD10 seems to have a compact single domain structure. The structures predicted by threading analysis to be most similar to PDCD10 include a six-helical bundle protein of no known function (1xwm), a five-helical bundle structure of vinculin (1rke), a four-helical bundle structure of the FAT domain of focal adhesion kinase-FAK (1pv3), and a four-helical bundle structure of the SNARE complex (1sfc) (Table 1). Most of these helical bundle proteins, similarly to PDCD10, are highly conserved throughout evolution [28]. Like Krit1 and OSM, these helical bundle proteins also function mainly in protein localization to the cytoskeleton and cellular membranes [35]–[37]. Based on this threading analysis, PDCD10 is likely to be a helical bundle protein composed of four to six helices. The compact structure of PDCD10 is uniquely distinct from Krit1 and OSM and suggests that PDCD10 may be an adaptor protein.

**Table 1:** Threading analysis using 3D-Jury.

3D-Jury's Score (Jscore)	PDB Hit	Name of Protein
43.67	1xwm_A	PhoU
39.11	1sum_B	PhoU protein homologue
36.89	1rke_A	Human vinculin
36.78	1h6g_A	α-catenin
34.22	1l7c_A	α-catenin
28.33	1pv3_A	FAT domain of FAK
27.89	1st6_A	Cytoskeleton protein
27.44	1k04_A	FAT domain of FAK
27.33	1ktm_A	FAT domain of FAK
24.44	1dow_A	α-catenin and β-catenin chimera
22.67	2bid_A	Pro-apoptotic protein BID
21.33	1ddb_A	Pro-apoptotic protein BID
21.33	1k40_A	Pro-apoptotic protein BID
21.00	1mfr_A	Ferritin
17.78	1k04_A	FAT domain of FAK
16.56	1wph_A	Metal binding protein
15.33	1sfc_D	SNARE complex

**Figure 1:**
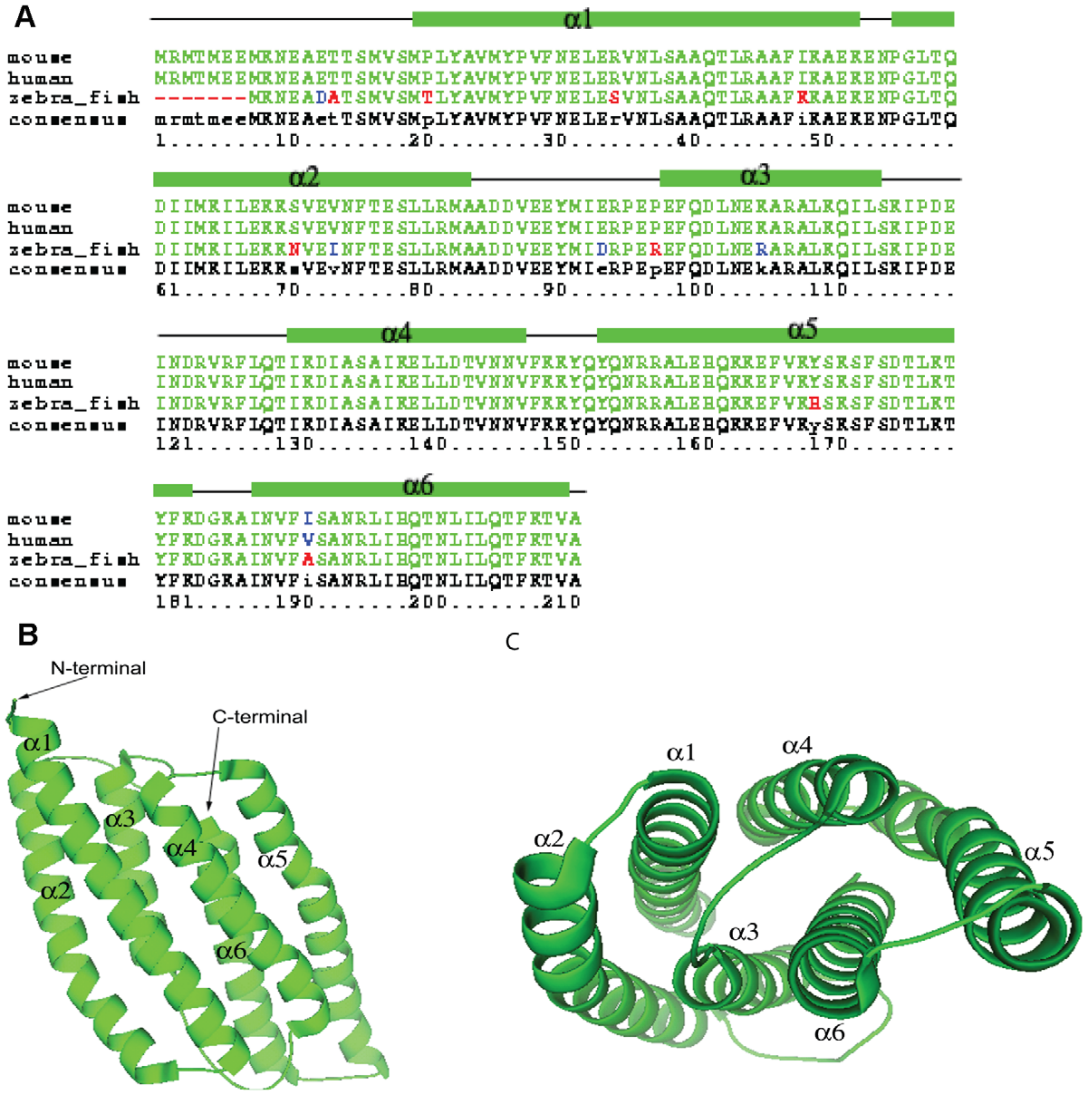
PDCD10 Model. A. Multiple sequence alignment of mouse, human, and zebra fish PDCD10 shows a highly conserved primary structure. B/C. A three dimensional model for PDCD10 shows a double heptad-repeat-hairpin structure.

**Figure 2:**
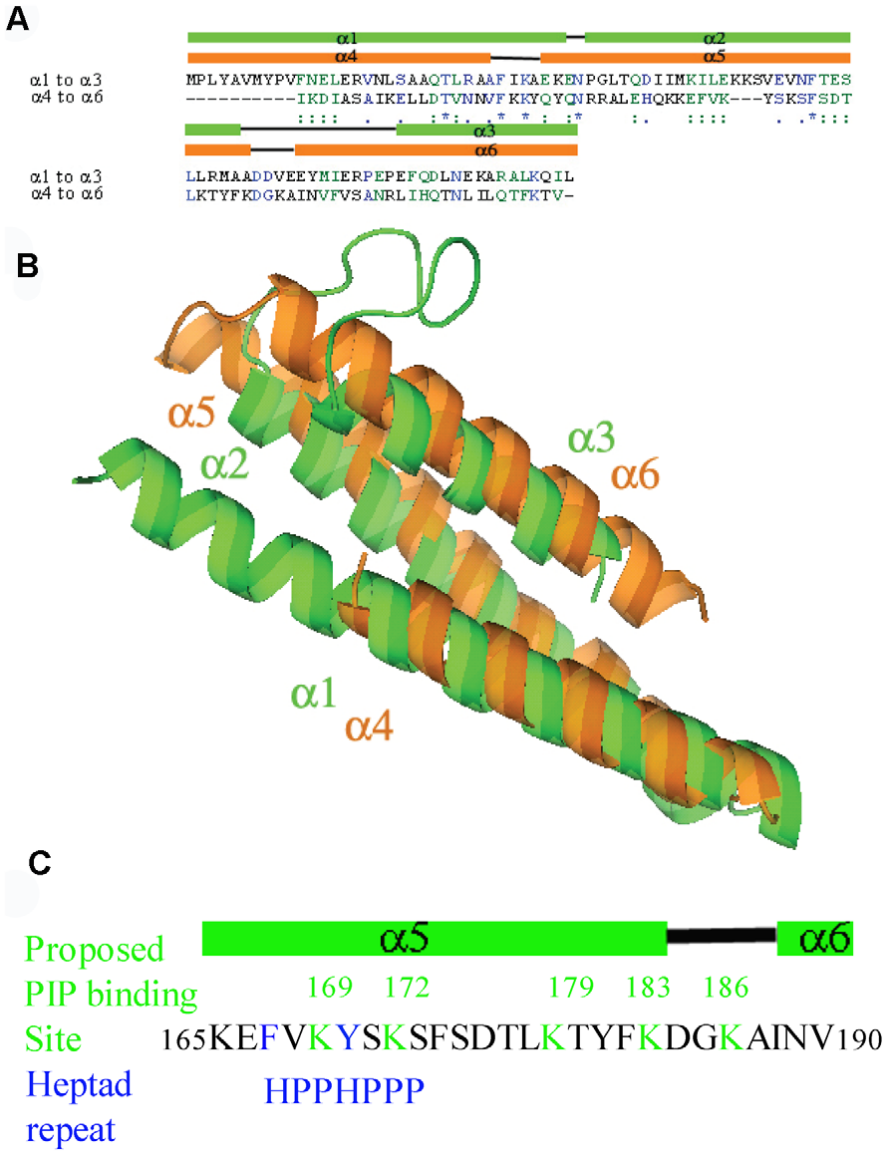
Heptad repeats and the amphipathic helix. A. Sequence alignment of the N-terminal and the C-terminal three-helix bundles. B. Superimposition of the N-terminal and C-terminal three-helix bundles shows that these two heptad-repeat-hairpin structures are related in the three dimensional structures. C. A diagram and primary sequence of the proposed amphipathic helix and the PIP binding site.

To further define the functional domain of PDCD10, multi-target modeling was used to generate a three dimensional model [38]–[45]. The model of PDCD10 shows a double heptad-repeat-hairpin structure and the potential interactive surfaces of PDCD10. The overall theoretical structure of PDCD10 is an α-helical structure with over 68% of the amino acids in α-helical conformations (Fig. 1A–B, supplemental structural model data). This structure is composed of a six-helix bundle formed by two three-helix bundles connected by a 15residue loop. The N-terminal three-helix bundle is composed of α1, α2, and α3, while the C-terminal three-helix bundle is composed of α4, α5, and α6. The interface between these two three-helix bundles forms a hydrophobic core, which is mediated mainly through hydrophobic residues located in α1–α4 and α3–α6. These two three-helix bundles are structural repeats known as heptad-repeats, a common random hydrophobic/hydrophilic repeat in coiled-coil structures [46]–[48] (Fig. 2A–B). The superposition of the two structural repeats of the three-helix bundle, α1–α3 with α4–α6, results in an r.m.s. deviation of about 5 Å over 85 equivalent Cα atoms (Fig. 2A–B). The major difference between these two repeats is that α1 is about 13 amino acids longer than α4. The other two helices, of the helical repeats α2/α4 and α3/α6, can be superimposed. Similar to the PhoU structures, superposition of the two repeats suggested that the protein sequences in these two repeats might be evolutionarily related [49] (Fig. 2A–B). Based on superimposition of all generated models, the C-terminal portion of the homology model appears to be more correct, while the N-terminal portion is less certain. The predicted ligand binding site is likely to be in the very end of the C-terminal portion. We therefore focused on the use of the homology model and ligand interaction in the C-terminal portion and did not attempt to predict other ligand interactions in the N-terminal portion.

**Figure 3:**
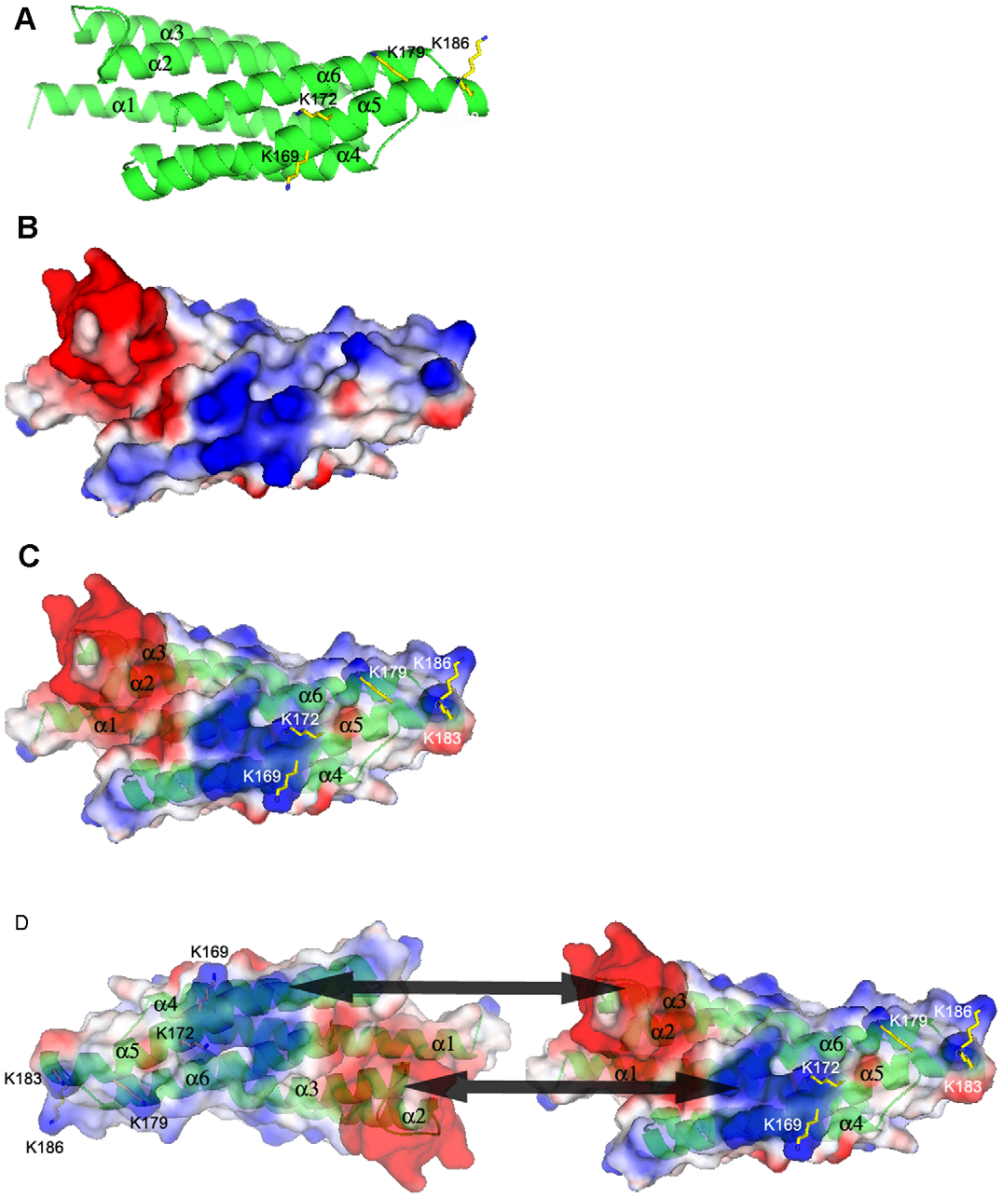
Proposed PtdIns(3,4,5)P_3_ binding site and dimeric interface. A. Ribbon model of PDCD10 showing the proposed PIP binding Lys residues. B. Surface-potential model of PDCD10 showing the surface positive potential charge area (blue) and negative potential charge area (red). C. Superimposition of the ribbon model and the surface-potential model. Note the proposed PIP binding Lys residues. D. Superimposition of the ribbon model and the surface-potential model shows the potential dimeric interface. Note that the dimeric interaction surfaces are mutually exclusive to the PIP binding site.

PDCD10 contains an unusually large number of highly flexible sidechains for a small protein: 21 lysine and 21 glutamate residues out of 212 total amino acids. Lysine and glutamate residue clusters are known to exhibit a highly variable surface due to flexible conformations [50]–[54]. This allows for structural flexilibity of the protein molecule that, in combination with hydrophobic areas, is often present in an interactive surface of a ligand or protein binding partner. The lysine residues in PDCD10 are conserved and located among conserved hydrophobic residues, in particular the C-terminal region (Fig. 1A, 2C). Our PDCD10 theoretical model suggests that this C-terminal region of PDCD10 may form an amphipathic helix that potentially binds to membrane phospholipids. Note that the majority of proteins with an amphipathic helix capable of binding to phospholipids often have lysine residues predominantly interspersed with hydrophobic residues, for example, the epsin ENTH domain (Protein Data Bank (PDB) code 1H0A), and the AP180/CALM ANTH domain (PDB code 1HFA) [55]–[57]. In the case of PDCD10, almost 10% of its amino acid content consists of scattered lysine residues. This distribution of lysine residues is seen in protein structures that interact with inositolphosphate ligands, including Drosophila melanogaster amphiphysin (PDB code 1URU) , endophilin-A1 BAR domain (PDB code 1ZWW), CIP4 (Cdc42-interacting protein-4), F-BAR domain (PDB code 2EFK), and IMD domain from IRSp53/missing-in-metastasis (PDB code 1Y2O) [58]–[62]. Lysine residues inside the bend of the cytoplasmic membrane indicates that the protein is sensing or stabilizing a highly curved membrane [57] .There are five conserved lysine residues in α5 and a flexible loop that connects this helix with the last helix, α6, including K169, K172, K179, K183, and K186 (Fig 2C). Surface potential analysis of the theoretical structure showed that these lysine residues form a cluster of positive charges interspersed with the hydrophobic residues (Fig 3A–C). These observations, in combination with our previous data showing that PDCD10 selectively binds to phosphatidylinostiol bis- and trisphosphates, but binds PtdIns(3,4,5)P_3_ with strongest affinity, led us to hypothesize that this area could be a potential amphipathic helix that may play a role in PtdIns(3,4,5)P_3_ binding [28].

### Defining PtdIns(3,4,5)P_3_ binding site of PDCD10

To determine whether the five lysines forming an amphipathic helix in our theoretical structure are important in PtdIns(3,4,5)P_3_ binding, we generated three mutants designed to eliminate important PtdIns(3,4,5)P_3_ binding lysine residues with two, three and five lysine-to-alanine mutations; Δ2KA (K169A, and K172A), Δ3KA K179A, K183A and K186A), Δ5KA (K169A, K172A, K179A, K183A, and K186A). The mutations in Δ2KA are located in the N-terminal portion of the amphipathic helix (α5), while the Δ3KA ones are in the C-terminal portion of the helix. The Δ5KA mutations would cover all possible lysines in the amphipathic helix. We expressed these recombinant mutant proteins along with wild-type PDCD10 with an N-terminal 6xHis-tag in bacteria as previously described [28]. We investigated the phospholipid binding potential of the PDCD10 recombinant proteins using Membrane Lipid Array and PIP-Arrays (Echelon, USA).

**Figure 4:**
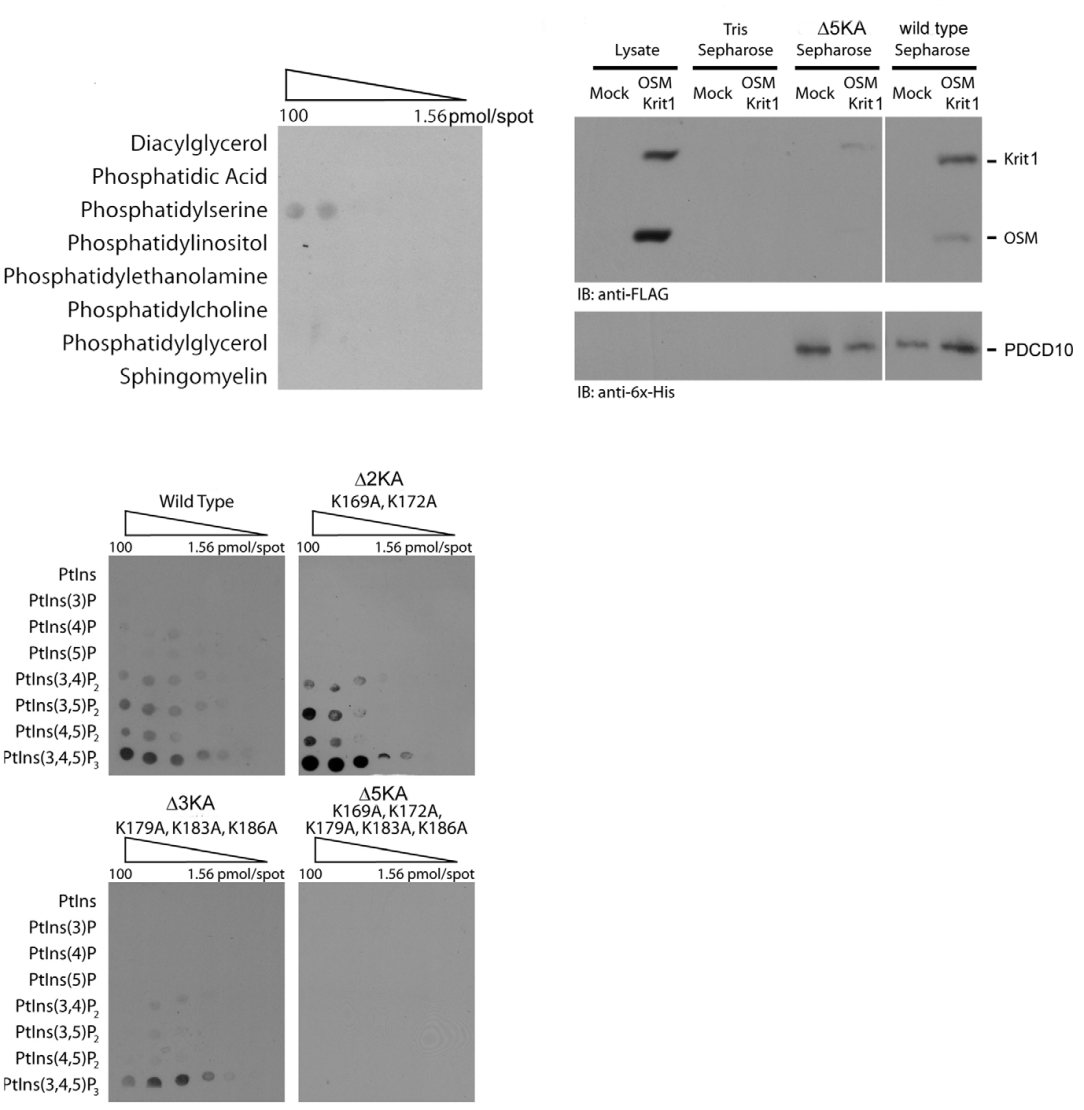
PDCD10 interactions with phospholipids and OSM. A. The Membrane Lipid Array shows that PDCD10 binds exclusively to phosphotidylserine with weak binding. B. PIP Arrays show the relative PIP binding affinity for the WT and three mutant PDCD10 proteins. C. Pull-down experiments, using recombinant purified WT and Δ5KA PDCD10 coupled to sepharose beads to pull-down FLAG-tagged overexpressed OSM and Krit1, showing that only the WT binds to the OSM-Krit1 complex.

In the Membrane Lipid Array, WT PDCD10 binds weakly but specifically to phosphoserine (Fig. 4A). However, in PIP-Arrays, WT PDCD10 selectively binds PtdIns(3,4,5)P_3_ with high affinity, and phosphatidyl bisphosphate (phosphatidylinositol 3,4 bisphophate (PtdIns(3,4)P_2_), phosphatidylinositol 3,5 bisphophate (PtdIns(3,5)P_2_), and phosphatidylinositol 4,5 bisphophate (PtdIns(4,5)P_2_) with moderate but lower affinity (Fig. 4B–C). We further screened for important lysine residues that are essential for PtdIns(3,4,5)P_3_ binding using mutant proteins. The Δ5KA mutation completely abolishes PtdIns(3,4,5)P_3_ and phosphatidylinositol bisphosphate binding, while the Δ2KA and Δ3KA mutants maintain similar phosphatidylinositol bisphosphates and PtdIns(3,4,5)P_3_ binding selectivity (Fig. 4A–C). Interestingly, the Δ3KA seems to have lower affinity to phosphatidylinostol bisphosphates than the WT and the Δ2KA. These results suggest that all five lysine residues play a critical role in PtdIns(3,4,5)P_3_ binding. However, there is some redundancy in the system and two to three lysines are sufficient for specific phosphatidylinositol bis- and tris-phosphate binding.

### OSM interaction with Δ5KA, a PDCD10 mutant lacking PtdIns(3,4,5)P_3_ binding residues

OSM (CCM2) interacts with Krit1 (CCM1) through a canonical PTB domain/NPXY motif interaction. Recently, we demonstrated that PDCD10 interacts with the OSM-Krit1 complex by interacting with OSM [28]. However, the mode of OSM-PDCD10 interaction is not known. The phosphatidylinositol bis- and tris-phosphate binding study indicated that there are five lysines located on the area of the α5 amphipathic helix that function as a PtdIns(3,4,5)P_3_ binding site (Fig. 2A,C). Based on our model, these five lysines reside in a surface exposure. For a relatively small protein like PDCD10, we expected that there may be redundancy in PtdIns(3,4,5)P_3_ and protein-protein interaction. We therefore further investigated if these lysines in the amphipathic helix (α5) are also involved in interactions with OSM. Recombinant purified WT and Δ5KA PDCD10 coupled to sepharose beads were used to pull-down FLAG-tagged overexpressed OSM and Krit1 (Fig. 4C). Interestingly, these pull-down experiments showed that the mutant fails to interact with OSM. These data suggests that the five lysine residues in this C-terminal region of PDCD10 are not only important in PDCD10-PtdIns(3,4,5)P_3_ interaction, but also important in PDCD10-OSM interaction.

### Secondary and tertiary structures of PDCD10

**Figure 5:**
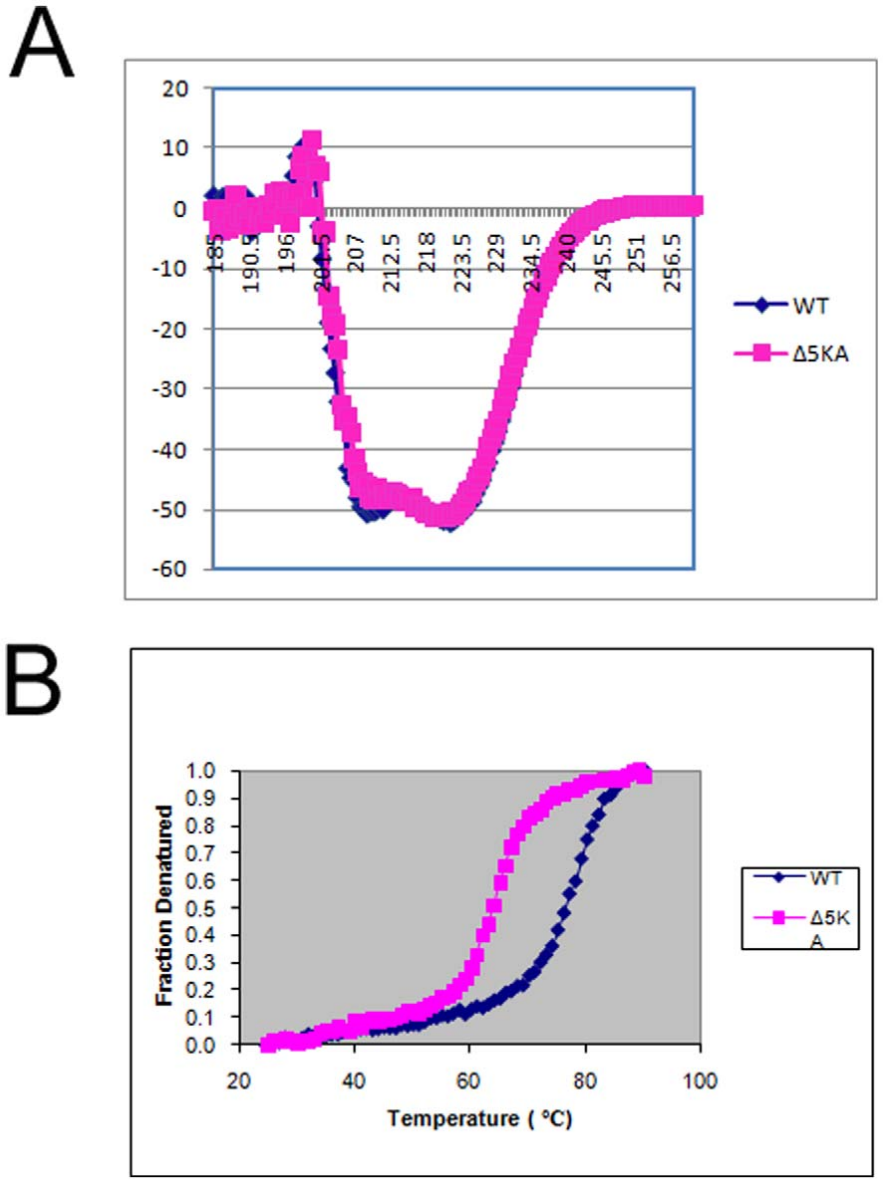
Circular dichroism and fraction denaturation. A. CD spectra of the WT and Δ5KA PDCD10 showing identical CD spectra. B. Fraction denaturation experiments show the lower Tm of Δ5KA compared to the WT.

To be sure that the mutation of lysines in Δ5KA does not alter the overall protein structure, we further examined the secondary and tertiary structures of the WT and Δ5KA mutant using purified recombinant proteins. Consistent with the modeling data, the circular dichroism (CD) spectra of the WT and Δ5KA appears to be of α-helical proteins. While the CD spectra of the WT and Δ5KA mutant were almost identical, the Tm of the WT is about 7°C lower than the one of the Δ5KA ((Fig. 5A–B). These results suggest that while the Δ5KA maintains similar secondary structure to the WT, it has lower molecular stability, perhaps as a result of five K-to-A mutations. In addition to examining the secondary structure, we used HPLC size-exclusion chromatography coupled with multi-angle laser light scattering (SEC-MALS) to determine the native molecular weights of the WT and Δ5KA PDCD10 proteins (Table 2). The results showed that both proteins are slightly larger than 50 KDa and therefore form a dimeric complex in solution. This dimeric form is the only species found in both WT and Δ5KA recombinant protein. The dimeric interface is therefore mutually exclusive from the phospholipid and OSM binding surface. The surface potential model of PDCD10 showed a highly negative charge area in the first trihelical heptad repeat and a highly positive charge area in the second trihelical heptad repeat (Fig 3D). The optimal docking area method indicates that these areas may play a role in the dimerization of PDCD10 [45].

**Table 2:** Determination of quaternary structure of wild-type and mutant Δ5KA ccm3 in solution using size exclusion chromatrography/multi angle laser light scattering (SEC-MAL).

	Determined Mw	Estimate Mw	Estimated Mw
		(Monomeric)	(Dimeric)
Wild-type	5.354 × 10^4^	2.852 × 10^4^	5.704 × 10^4^
Δ5KA	5.054 × 10^4^	2.741 × 10^4^	5.482 × 10^4^

### Cellular co-localization of PDCD10 and membrane-bound constitutively-active phospholinositol-3-kinase (p110-CAAX)

**Figure 6:**
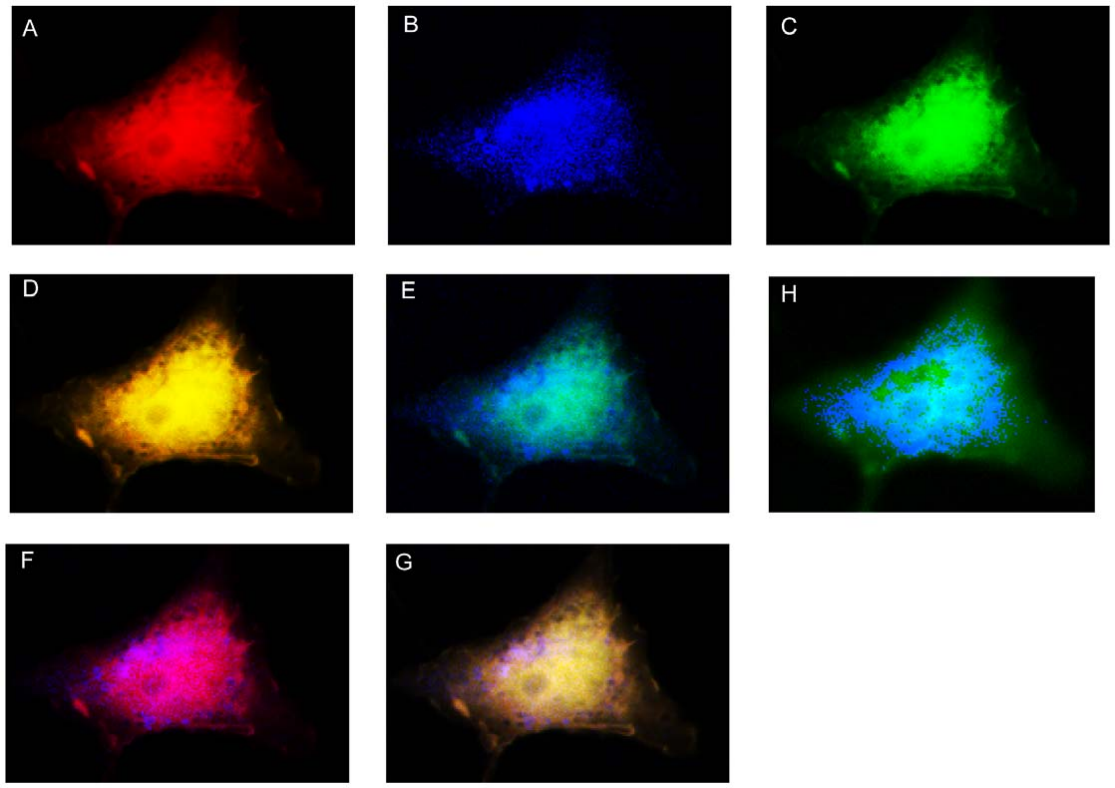
Co-localization of PDCD10 and p110-CAAX. Overexpression of the WT and Δ5KA PDCD10 together with p110-CAAX shows that WT PDCD10 and p110-CAAX colocalize to the membrane while the Δ5KA stays only in the cytoplasm. A. WT in Mcherry. B. Δ5KA in CFP. C. p110-CAAX in FITC. D. A composite picture of WT (Mcherry) and p110-CAAX (FITC). E. A composite picture of Δ5KA (CFP) and p110-CAAX (FITC). F. A composite picture of WT (Mcherry) and Δ5KA (CFP). G. A composite picture of WT (Mcherry), p110-CAAX (FITC), and Δ5KA (CFP). H. A composite picture of Δ5KA (CFP) and p110-CAAX (FITC). A light cyan mask was used to enhance the visualization of Δ5KA localization.

The fact that PDCD10 seems to have the highest affinity with PtdIns(3,4,5)P3 led us to believe that PDCD10 may function with phospholinositol-3-kinase (PI3K). PI3K is a master kinase that catalyzes the phosphorylation of PtdIns(4,5)P_2_ to produce PtdIns(3,4,5)P_3_. PI3K catalyzes PtdIns(3,4,5)P_3_ formation upon activation by growth factors such as VEGF [29]. We used the catalytic subunit of PI3K (lacking a regulatory domain) with a COOH-terminal plasma membrane targeting sequence to produce a PI3K construct that is constitutively active (p110-CAAX) [63]–[65]. When WT PDCD10, Δ5KA, and p110-CAAX were co-transfected, WT PDCD10 and p110-CAAX were co-localized, while the Δ5KA was not (Fig 6). This experiment confirms our biophysical data and suggests that PDCD10 binds to PtdIns(3,4,5)P_3_ and may function in the PI3K signaling pathway.

## Discussion

CCM is a unique example of a genetic disorder that results from the dysfunction of three non-catalytic signaling proteins. Krit1-OSM-PDCD10 form a complex in the cell and CCM lesions appear to result from a loss of integrity of this complex. While Krit1 and OSM proteins both have known structural domains and functionally link to several important signaling pathways, including integrin signaling, the p38 MAP kinase pathway, and RhoA-rho kinase regulation, PDCD10 lacks known structural domains. PDCD10 was thought to relate to apoptosis; however, there is no clear apoptotic pathway known. Defining a functional domain of PDCD10 is therefore important in learning how this protein functions and contributes to CCM development. Combining molecular modeling and site-directed mutagenesis, it appears that PDCD10 is a six helical bundle protein composed of a two heptad repeat, and α5 is an important amphipathic helix housing five lysine residues essential for PtdIns(3,4,5)P3 binding. Unlike phosphatidylinositol specific domains, for instance C1, PH, PX and FYVE, amphipathic helices often only demonstrate high specificity to certain phosphatidylinositol molecules under specific circumstances, such as when the membrane area possesses a highly curved region [57]. We speculate that this may be the case for PDCD10 since there seem to be multiple lysine residues beyond the amphipathic helix similar to some phosphatidylinositol binding proteins that use these outside amphipathic helix lysine residues to interact with the curved membrane region [57]–[62]. We further show that the Δ5KA, a mutant lacking these five important K residues, does not bind to PtdIns(3,4,5)P_3_ *in vitro*. While the WT and Δ5KA both appear to have the same helical secondary/tertiary structure, and are dimeric in solution, the Δ5KA does not bind OSM. In addition, when WT PDCD10 and Δ5KA are co-expressed with p110-CAAX, a membrane-bound/constitutively active PI3K, WT is co-localized with p110-CAAX at the plasma membrane. However, Δ5KA stays in the cytoplasm. The results suggest that PDCD10 may function with PI3K.

A recent study shows that PDCD10 functions in VEGF-dependent translocation of vascular endothelial growth factor receptor 2 (VEGFR2) and further, suggests that the C-terminal portion of PDCD10 is important in PDCD10-VEGFR2 interaction (66). VEGF is an upstream regulator of PI3K. It is therefore plausible that PDCD10 may play a role in VEGF-PI3K signaling. Interestingly, colocalization of PDCD10 and PI3K in our study is almost identical to the colocalization of PDCD10 and VEGFR2 [66]. It is possible that this colocalization is a result of PDCD10 and VEGFR2 interactions. It was shown that PDCD10 interacts with VEGFR2 through its C-terminal region [66]. This region overlaps with the PtdIns(3,4,5)P_3_ binding where we located the five PtdIns(3,4,5)P_3_ binding lysines. These lysines are also important in PDCD10-OSM interaction. Furthermore, genetic studies demonstrate that this region is predisposed to frameshift mutations that often cause early termination of the protein resulting in CCM [17]. We therefore speculate that PDCD10-OSM and PDCD10-VEGFR2 interactions may be regulated by the availability of PtdIns(3,4,5)P_3_ generated by PI3K.

**Figure 7:**
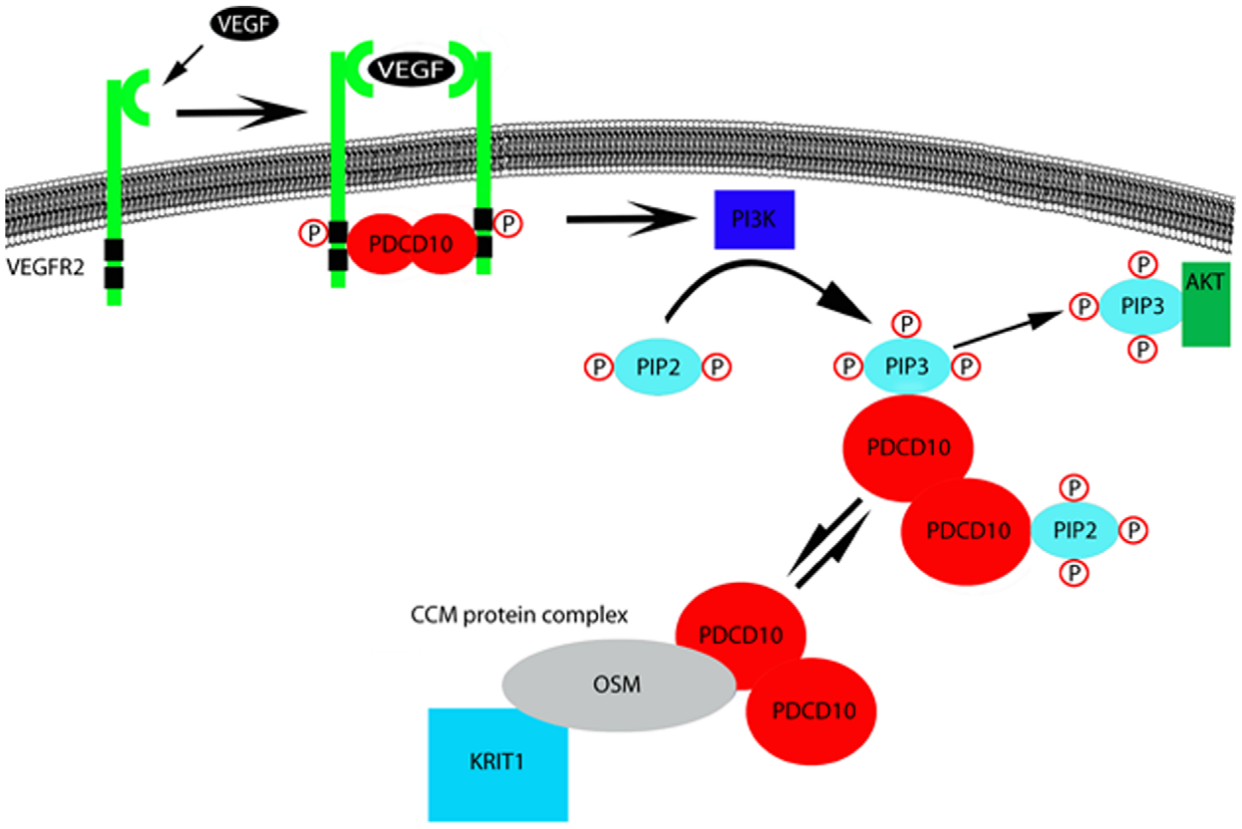
Proposed PDCD10 signaling model.

Based on our findings and recent studies, we composed a signaling model for PDCD10 (Fig 7). We propose that PDCD10 functions closely with VEGFR2 and PI3K. Upon activation of VEGFR2 by VEGF, VEGFR2 binds to dimeric PDCD10, translocates to the membrane, and becomes activated. As a result of VEGFR2 activation, PI3K is activated and favors the catalysis of PtdIns(3,4)P_2_ or PIP_2_ to PtdIns(3,4,5)P_3_ or PIP_3_ (Fig 7). PtdIns(3,4,5)P_3_ goes on to bind PDCD10 and AKT. There may be an equilibrium between PDCD10-PtdIns(3,4,5)P_3_ and PDCD10-OSM/Krit1, as well as a equilibrium between PDCD10-PtdIns(3,4,5)P_3_ and AKT-PtdIns(3,4,5)P_3_. PtdIns(3,4,5)P_3_ and OSM seem to have the same interactive site on the PDCD10 dimer, and it is possible that VEGFR2 may also share the same binding site. It is therefore plausible that PDCD10 may regulate the function of these three important signaling molecules at the same time using simple chemical equilbrium.

We propose that CCM development may result from the dysregulation of the VEGF/PI3K signaling pathway through PDCD10- PtdIns(3,4,5)P_3_ interaction. Future experiments will need to be completed to define the role of PDCD10 in PI3K signaling. PI3K can be activated by several growth factors, including VEGF. Characterizing the role of PDCD10 during PI3K activation by VEGF, as well as downstream effectors such as AKT1, will be important in understanding CCM development and how we may treat this condition by modulating the activity of these kinases. Linking the role of PDCD10 in the VEGF/PI3K pathway to other CCM proteins will elucidate the individual roles of CCM proteins, as well as the CCM protein complex . We recently showed that OSM plays a role in regulating the degradation of the small GTPase RhoA and that the CCM endothelial phenotype can be rescued with knockdown or inhibitors of ROCK-RhoA effectors [67]. Interestingly, activation of PI3K can also increase RhoA activation [68]. Further experiments will also be needed to determine if PDCD10 functions in concert with OSM and Krit1 in signaling the regulation of RhoA and PI3K.

## Materials and Methods

### Generating a three-dimensional model of PDCD10 using multiple templates

#### PDCD10 structure prediction

The following protein structure prediction protocol was recently tested in the critical assessment of techniques for protein structure prediction (CASP8; <http://predictioncenter.org>). Not only did this method rank as one of the best, but all predictions with normalized RAPDF scores better than −55 (described below) were within 3.5 Ångstrom root mean squared deviation of the corresponding experimental PDB structure, indicating correct overall topology. Additionally, the refinement method consistently improved initial models, in some cases resulting in models within the accuracy of the corresponding experimental structures themselves.

#### Initial models

The initial comparative modeling templates were identified using the secondary structure enhanced profile-profile threading alignment (LOMETS) and the four part iterative threading assembly refinement protocol of I-TASSER [38]–[39]. Ten different protein structure prediction servers were sampled, each producing at least five models. All those conforming, at least in part, to the selected templates (1kil, 1s35, 1sum, 1txd, 2boq, 2i0m, 2of3) were used in further analysis, along with five models produced by PROTINFO [40] from the two full length templates (1sum, 2i0m).

Iterations of ENCAD energy minimization and SCWRL3.0 sidechain optimization were applied to increase the sampled conformational space between models, such that variation and coverage were sufficient for clustering analysis [41]–[42]. With the resultant model set, an iterative density calculation was applied, which cycles between a cluster density calculation and removal of outliers. Centroids for the five largest sub-clusters were then taken as the five input models for refinement.

#### RAPDF and consensus based constraint selection

From the five initial models, a set of consensus interatomic distances was derived as all atom-atom pair distances which occur within a 0.5 Ångstrom window for at least four of the five models. A residue specific all atom probability discriminatory function (RAPDF) was used to score the consensus distances. Here the philosophy was that the probabilities derived from a Bayesian analysis of distances observed in a structurally non-redundant database of experimentally derived protein models versus random are likely to be useful to build models similar to the native state protein conformation [43]. A batch-by-batch method compiled the final distance set, starting with the consensus distances having the highest RAPDF scores, resulting in a single interatomic distance for each possible residue pair in the protein. Each distance was weighted for importance in model building by the RAPDF score and whether the distance was observed in four or all of the five input models. Finally, three constraint sets were built using different maximal distance cutoffs (12 Å, 16 Å, 20 Å).

#### Model building and final selection

The three constraint sets were each used in fifty rounds of CYANA restrained torsion angle dynamics simulations, for which a Ramachandran plot-like distribution of torsion angles observed in the non-redundant structure database was used to prescribe probabilities for torsion angles [44]. Each round produced twenty all-atom models, with a total of three thousand conformations created. Half of the conformations were filtered by sequentially applying RAPDF, a van der Waals energy term, the hydrophobic compactness factor, and an electrostatics term. The resulting models were minimized by ENCAD and SCWRL3.0, and subsequently the iterative density calculation was again applied to cyclically remove outliers and re-cluster, and finally select the centroid for each of the five largest clusters. A new set of interatomic distances were obtained from the resulting five models, and used in a second round of consensus modeling which produced the final tertiary structure predictions.

#### Homodimer interface site prediction

Protein-protein interface sites were identified by applying the optimal docking area method (ODA; <http://www.molsoft.com/oda.html>), which segregates surface patches and applies atomic desolvation calculations parameterized with octanol/water transfer experiments adjusted to protein-protein interactions [45].

### Examination of phospholipid and OSM binding using recombinant PDCD10 proteins

#### Membrane lipid and PIP arrays

Production of recombinant PDCD10 protein was described previously [28]. Full-length murine PDCD10 was amplified using PCR from a mouse fibroblast cDNA library and cloned into pMCSG7-His. Recombinant murine 6xHis-PDCD10 was expressed in BL21 cells and purified by nickel affinity chromatography. Three mutants were generated using site-directed mutagenesis (QuikChange, Stragetagene) including two K-to-A mutations (Δ2KA:K169A, and K172A), three K-to-A mutations (Δ3KA:K179A, K183A and K186A), and five K-to-A mutants (Δ5KA: K169A, K172A, K179A, K183A, and K186A). Similar to the WT protein, mutant proteins were expressed with N-terminal 6xHis-tag in BL21DE3 cells. Membrane lipid arrays and PIP arrays were purchased from Echelon Biosciences. Membranes were blocked in 0.1% ovalbumin in TBS-T for one hour then incubated with 1 µg/ml of recombinant protein for two hours. After washing unbound protein using TBS-T, bound protein was detected by immunoblotting with an anti-His antibody (Santa Cruz Biotechnology).

#### Pull-down experiments

For pull-down experiments, vectors encoding FLAG-tagged Krit1 and OSM were transfected into HEK293 cells (the American Type Culture Collection (ATCC) Rockville, MA, USA) using lipofectamine. Twenty-four hours after transfection, cells were harvested in a non-ionic detergent containing lysis buffer, and total protein concentration was determined by the Bradford method. 10 µg His-tag recombinant WT and mutant Δ5KA of PDCD10 were bound to CNBr-activated sepharose beads (GE Biosciences) and incubated with 500 µg of cell lysate for 16 hours at 4°C. Beads were collected by centrifugation and washed 3× with lysis buffer. Washed beads were then mixed with 30 µl 2× SDS-PAGE buffer and analyzed on a 10% polyacrylamide gel. FLAG-tagged and His-tag proteins were detected by immunoblotting similarly as previously described [28].

### Examination of the tertiary and secondary structures of PDCD10

#### Size-exclusion chromatography-multi angle laser light scattering (SEC-MALS)

0.2 to 0.4 mg/ml of purified His-tag recombinant WT and Δ5KA PDCD10 were dialyzed in 10 mM phosphate buffer, pH 7.0 and 500 mM NaCl. The absolute molecular weight of each protein was determined using high-performance size-exclusion chromatography (SEC-MALS) composed of Wyatt DAWN EOS light scattering instrument interfaced to an Amersham Biosciences Akta FPLC, Wyatt Optilab refractometer, and Wyatt dynamic light scattering module at the UNC-CH Macromolecular Interaction Facility using methods similar to ones previously described [69]–[70].

#### Circular dichroism (CD), and thermal denaturation studies

CD and thermal denaturation experiments were conducted using an Applied Photophysics PiStar-180 CD spectropolarimeter. WT or Δ5KA PDCD10 (0.15 mg/ml; in 10 mM phosphate buffer, pH 7.0) was used. CD data was collected for each protein and ranged in wavelength from 185 to 260 nm. For the thermal denaturation experiments, the temperature was increased from 25°C to 90°C while monitoring at 222 nm. Plots of fraction denatured versus temperature were produced by defining the upper and lower temperature baselines as 0 and 100%, respectively.

### Examination of PDCD10-PI3K colocalization in cell

#### Cell culture, immunostaining, and co-localization analysis

COS7 cells (the American Type Culture Collection (ATCC) Rockville, MA, USA) were maintained in DMEM (LifeTechnologies) with 10% FBS, 100 U/ml penicillin, and 100 µg/ml streptomycin at 37°C with 7% CO_2_. Recombinant human IL-1β was obtained from PeproTech. Polyclonal anti-Myc antibody was obtained from Santa Cruz Biotechnology and FITC goat anti-rabbit antibody was obtained from Invitrogen. pBabe-p110-CAAX-Myc was generously provided by Dr. Channing Der (University of North Carolina).

COS7 cells were transfected with WT PDCD10-mCherry, Δ5KA-ECFP, and p110-CAAX-Myc, and were plated on 22 mm square glass coverslips in a 6-well plate using Lipofectamine2000 as recommended by the manufacturer (Invitrogen). After 24 hours, the cells on coverslips were fixed for 20 minutes with 4% paraformaldehyde in PBS at 25°C. Permeabilization was conducted using 0.1% Triton X-100 in PBS for 10 min. Nonspecific binding was blocked by incubation of coverslips for 1 h in 10% goat serum in PBS. The coverslips were incubated with polyclonal anti-Myc antibody (Invitrogen) for 1 h and washed with PBS. Bound primary antibodies were visualized by incubation with FITC goat anti-rabbit antibody for 1 h, washed, and mounted on glass slides. Imaging was performed using a Zeiss Axiovert 200 M inverted microscope with a 125-W xenon arc lamp (Sutter Instrument Company, Novato, CA), digital CCD camera (CoolSNAP HQ, Roper Scientific, Tucson, AZ), and Slidebook 5.0 software (Intelligent Imaging Innovations, Denver, CO). An objective (63× Oil 1.25-numerical aperture, Plan-Neofluar, Zeiss) was coupled with immersion oil to the bottom face of glass coverslips. The images were obtained at 50 and 10 ms exposure with 2 × 2 binning, respectively. For section analyses, the background images from three planes were taken for each of the three channels (CFP [a band-pass excitation filter of 436/20 nm, a 455DCLP band beamsplitter, and a band-pass emission filter of 480/40 nm], YFP [a band-pass excitation filter of 500/20 nm, a 515DCLP band beamsplitter, and a band-pass emission filter of 535/30 nm], and Cy5 [a band-pass excitation filter of 620/60 nm, a 660DCLP band beamsplitter, and a band-pass emission filter of 700/75 nm]; Chroma). The three planes were deconvolved using the nearest neighbor's algorithm.
